# Bioresorbable vascular stents and drug-eluting stents in treatment of coronary heart disease: a meta-analysis

**DOI:** 10.1186/s13019-020-1041-5

**Published:** 2020-01-28

**Authors:** Le Ni, Hao Chen, Zhurong Luo, Yunqiang Yu

**Affiliations:** The 900th Hospital of PLA Joint Logistics Support Force, 156 West Second Ring Road, Fuzhou City, Fujian Province China

**Keywords:** Bioresorbable vascular stents, Drug-eluting stents, Coronary heart disease, Meta-analysis

## Abstract

**Objective:**

To compare the efficacy and safety of bioresorbable vascular stents (BVS) and drug-eluting stents (DES) in coronary heart disease.

**Methods:**

The full text of clinical studies involving BVS and DES was retrieved in PubMed, Springer, EMBASE, Wiley-Blackwell, and Chinese Journal Full-text Database. Review Manager 5.3 was used for meta-analysis to evaluate the risk of target lesion failure, stent thrombosis and cardiac death in BVS and DES.

**Results:**

Finally, 10 studies with 6383 patients were included in the meta-analysis. Compared with DES group, BVS group had significantly increased risk of target lesion failure (OR = 1.46, 95%CI 1.20–1.79, *P* = 0.0002; P _Heterogeneity_ = 0.68, I^2^ = 0%), stent thrombosis (OR = 2.70, 95%CI 1.57–4.66, *P* = 0.0003; P _Heterogeneity_ = 1.00, I^2^ = 0%) and cardiac death (OR = 2.19, 95%CI 1.17–4.07, *P* = 0.01; P _Heterogeneity_ = 0.93, I^2^ = 0%).

**Conclusion:**

This study shows that DES is a safer treatment than BVS for coronary revascularization.

## Background

Coronary heart disease (CHD), has been a leading cause of morbidity and mortality in the world [1, 2]. The prevalence of CHD is increasing year by year and patients tend to be younger [3, 4]. Percutaneous coronary intervention (PCI) with stents is a common treatment strategy for CHD patients with significant stenosis of coronary arteries (> 70%). Nowadays, drug-eluting stents (DES) are widely used in PCI. Compared with previous bare metal stents, the obvious improvement of DES is the carriers of anti-proliferation drugs [5]. The drug carriers of DES are mainly polymer coatings, which are designed to carry enough drug dosage and can effectively control the decomposition, diffusion and release of paclitaxel or other drugs.

Bioabsorbable vascular stent (BVS) is a type of newly invented stent and theoretically have a number of potential benefits [6, 7]. First, the occlusion of coronary artery can be opened by BVS implantation. Second, after being absorbed, BVS can restore normal vasomotion and endothelial function. In past several years, several clinical studies have been conducted to compare the efficacy of BVS with DES in parameters like target lesion failure. However, the outcomes were inconsistent and remain to be identified [8–11].

To establish the clinical efficacy of BVS, we conducted this meta-analysis of available randomized controlled trails (RCT) and clinical prospective studies comparing BVS and DES in CHD.

## Materials and methods

### Search strategy

The comparison between BVS and DES was comprehensively analyzed. Articles from inception to October 2018 were searched from PubMed, Springer, EMBASE, Wiley-Blackwell, and Chinese Journal Full-text Database. Systematic reviews and meta-analysis were conducted.

Two members of our team searched for articles independently using the following keywords: (1) bioresorbable stents OR BVS; (2) drug-eluting stents OR DES; (3) coronary heart disease OR CHD. All these terms are assembled with the connection symbol “and” to search the database for related articles. In order to obtain more relevant research and higher accuracy, the reference list of each article retrieved were also reviewed.

### Citation selection

All articles after the first screening were further selected by two other authors. The titles and abstracts of these articles are independent and carefully screened. Then, if the research may be relevant, full-text research will be obtained.

The following inclusion criteria must be met in the citations included in this study:

1. A randomized control trial study or a controlled clinical trial study;
2. Comparison of the treatment between BVS and DES;
3. Availability of full text.

Exclusion criteria:

1. Observational studies;
2. Studies on other treatments other than BVS or DES;
3. Studies lacking outcome measures or comparable results.

Finally, the two authors jointly identified included articles. They examined whether the study met the above requirements. If there was any difference or no agreement was reached, the third investigator helped to make the decision.

### Data extraction

Two reviewers read the full text and extracted the relevant data of each study into the coding table in Microsoft Excel software. The characteristics extracted in this study included the first author’s name, publication year, year of onset, sample size (bioresorbable/drug-eluting), age range of patients and outcome parameters. The parameters were about target lesion failure, stent thrombosis and cardiac death in BVS and DES.

### Statistical analysis

Meta-analysis was performed by Revman 5.3 (Cochrane Collaboration, 2014) to assess differences in clinical efficacy between BVS and DES and to assess publication bias. Q statistics reflect the level of heterogeneity. When the heterogeneous I^2^ statistic was greater than 50% reflecting moderate or high heterogeneity, a random effect model was used, otherwise a fixed effect model was deployed.

We also performed a bias analysis of each study with the following criteria: (1) random sequence generation, (2) allocation concealment, (3) blinding of participants and personnel, (4) blinding of outcome assessment, (5) incomplete outcome data, (6) selective reporting, and (7) other bias. In our studies, all parameters are two variables and the corresponding risk of 95% confidence interval (CIS) is calculated (RR). Funnel plots together with Egger tests were also applied to assess possible publication bias. *P* value < 0.05 was considered that statistically significant was observed.

## Results

### Search results

**Fig. 1:**
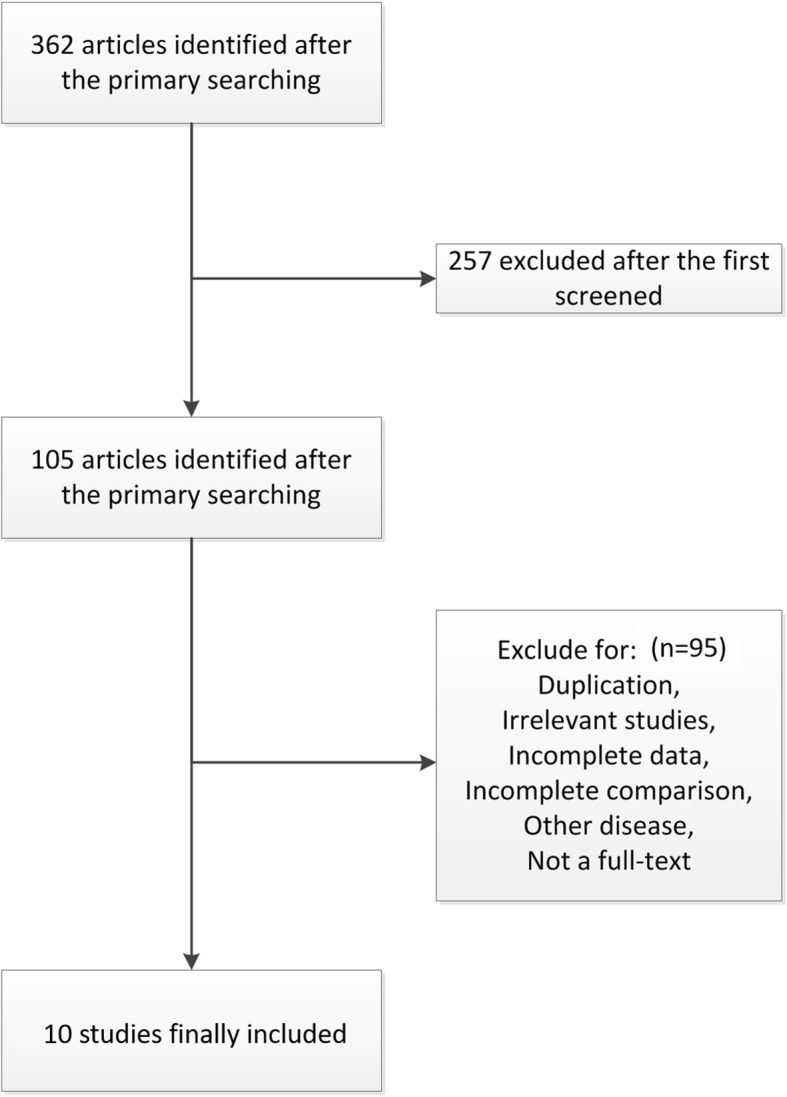
Flow diagram of the study identification, inclusion and exclusion

A total of 362 related articles were found in the preliminary search of electronic database. After a thorough review, 10 papers eventually met all inclusion criteria [8–17]. The other 352 articles were excluded due to duplication, article types, irrelevant studies, no control groups, incomplete data or comparisons. Figure 1 is a flowchart of identification, inclusion and exclusion, reflecting the search process and the reasons for exclusion.

### Characteristics of included studies

Table 1 lists the first author’s name, year of publication, sample size (bioresorbable/drug-eluting), age range of patients, and outcome parameters for each study. All these articles were published from 2010 to 2018. The sample size is between 35 and 2604. At last, 6383 patients with coronary heart disease, including 3573 in BVS group and 2810 in DES group were included in our meta-analysis.

**Table 1:** Characteristic of the included studies

Study	Year	Language	Country or Region	Age range (mean)	Groups	n	Years of onset
Abizaid	2016	English	Brazil	62 ± 10	BVS	63	Nobember 2011 to JUne 2012
					DES	63	
Brugaletta	2012	English	Netherlands	60.5 ± 9.1	BVS	17	January 2005 to December 2010
					DES	18	
Ellis	2015	English	USA	63.5 ± 10.6	BVS	1322	NA
					DES	686	
Huang	2018	English	Taiwan	56.7 ± 3.4	BVS	112	August 2012 to December 2014
					DES	125	
Kim	2018	English	Korea	61.2 ± 4.1	BVS	232	January 2004 to January 2012
					DES	232	
Kim2	2018	English	Korea	64.3 ± 6.7	BVS	71	November 2011 to December 2015
					DES	87	
Puricel	2015	English	Switzerland	64.1 ± 5.9	BVS	80	January 2010 to January 2014
					DES	80	
Sato	2016	English	Germany	58.8 ± 10	BVS	45	January 2010 to December 2014
					DES	45	
Serruys	2015	English	Netherlands	61.2 ± 10.0	BVS	335	November 2011 to June 2013
					DES	166	
Stone	2018	English	USA, Germany, Australia, Singapore, and Canada	63.1 ± 10.1	BVS	1296	August 2014 to March 2017
					DES	1308	

*NA* None available

### Quality assessment

The deviation table in the Review Manager 5.3 tutorial is used to assess the risk of each study by applying the criteria for evaluating design-related deviations. The risk of bias in this study is listed in Table 2. Participants and respondents had a high risk of blindness due to significant differences between bioresorbable group and drug-eluting group.

**Table 2:** The risk of bias table in this study

	Abizaid	Brugaletta	Ellis	Huang	Kim	Kim2	Puricel	Sato	Serruys	Stone
Random sequence generation	low	not	high	not	low	low	high	high	low	high
Allocation concealment	low	low	high	high	high	high	low	low	low	high
Blinding of participants and personnel	high	high	high	high	high	high	high	high	high	high
Blinding of outcome assessment	not	low	high	high	low	low	low	low	not	high
Incomplete outcome data	not	low	high	low	not	not	low	low	low	high
Selective reporting	high	high	high	not	low	low	low	not	low	high
Other bias	not	low	high	not	low	low	not	not	low	high

Note: in this table, “low” stands for “low risk”, “high” stands for “high risk”, “not” stands for “not clear”

### Results of meta-analysis

#### Meta-analysis about target lesion failure

**Fig. 2:**
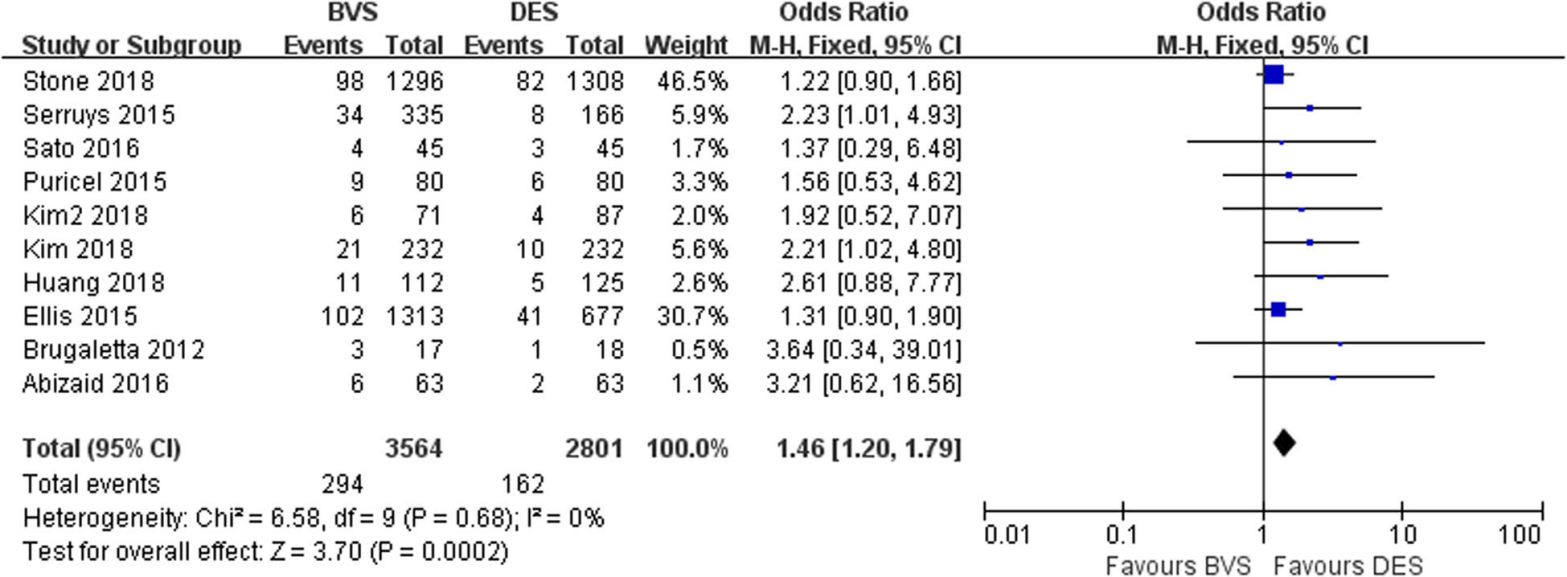
A forest plot for target lesion failure in BVS and DES groups

Ten studies involved target lesion failure. All 10 studies showed statistically significant differences in target lesion failure between BVS and DES. The meta-analysis suggested that the target lesion failure of the BVS group was significantly higher than that of DES group with no heterogeneity among studies (OR = 1.46, 95% CI 1.20–1.79, *P* = 0.0002; P _Heterogeneity_ = 0.68, I^2^ = 0%; Fig. 2).

#### Meta-analysis about the stent thrombosis

**Fig. 3:**
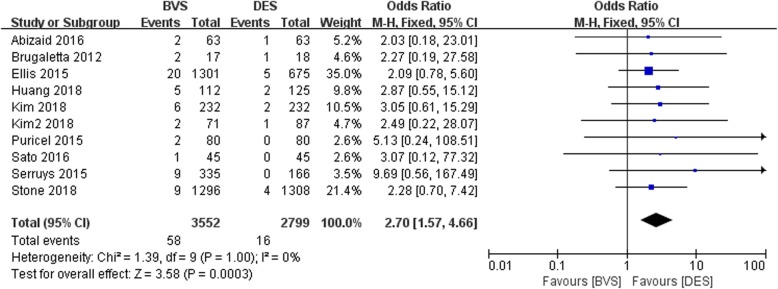
A forest plot for stent thrombosis in bioresorbable and drug-eluting groups

The forest plot for meta-analysis about the stent thrombosis was presented in Fig. 3. The results demonstrated that the stent thrombosis in BVS group was significantly higher than that of DES group with no heterogeneity among studies (OR = 2.70, 95%CI 1.57–4.66, *P* = 0.0003; P _Heterogeneity_ = 1.00, I^2^ = 0%; Fig. 3).

#### Meta-analysis about the cardiac death

**Fig. 4:**
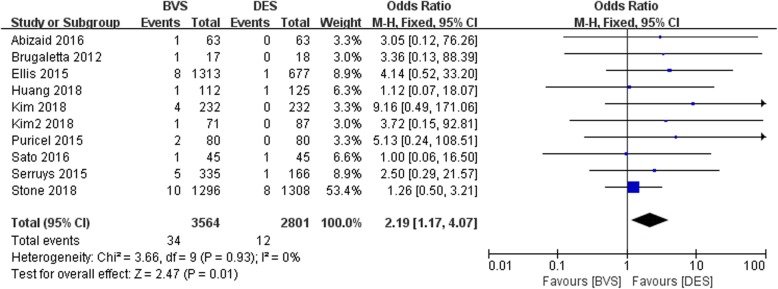
A forest plot for cardiac death in bioresorbable and drug-eluting groups

All included studies about the cardiac death was shown in Fig. 4. The overall result indicated that the cardiac death in BVS was significantly higher than that of DES group with no heterogeneity among studies (OR = 2.19, 95%CI 1.17–4.07, *P* = 0.01; P _Heterogeneity_ = 0.93, I^2^ = 0%; Fig. 4).

### Bias analysis

**Fig. 5:**
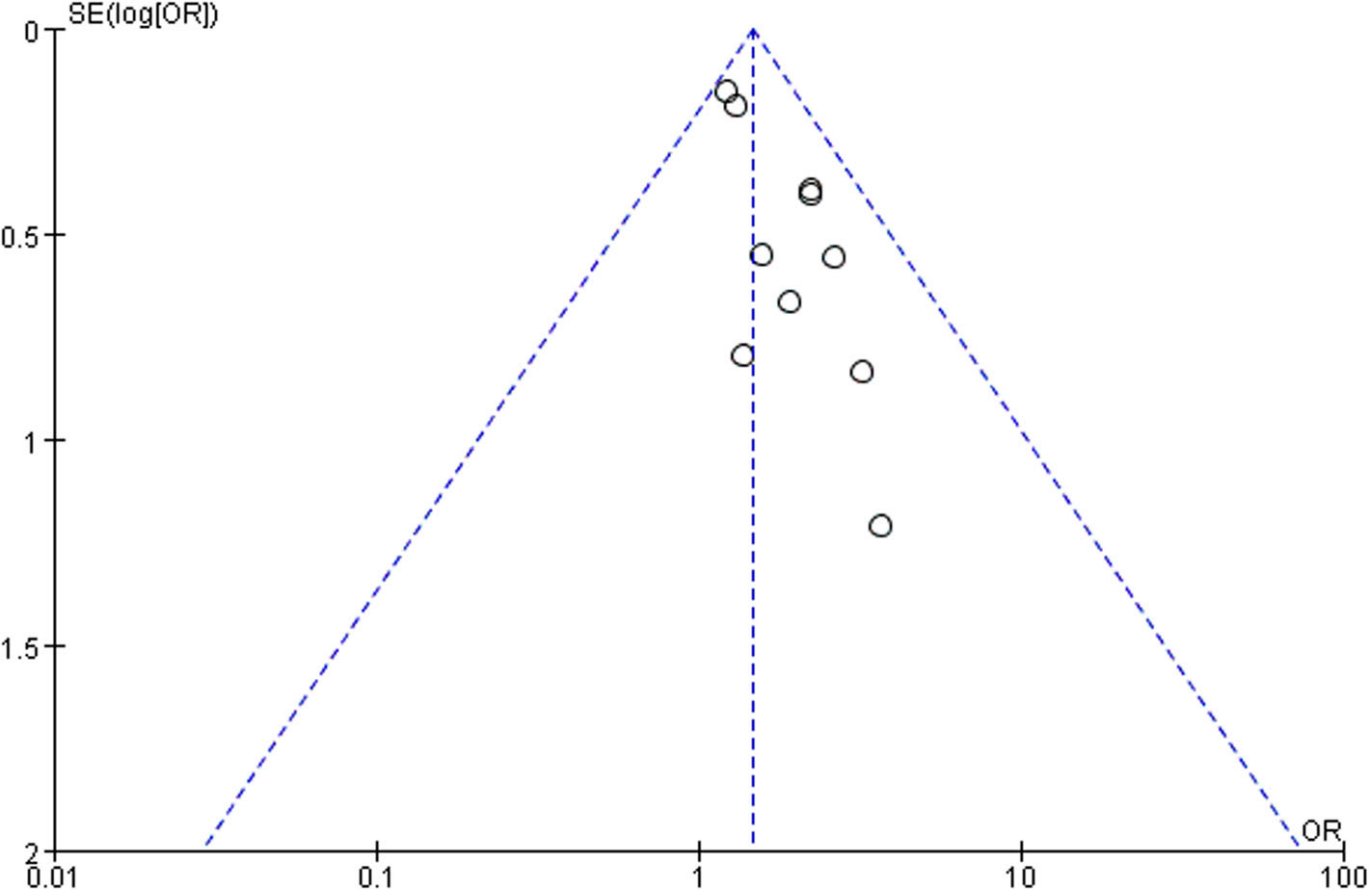
Begg’s funnel plot of publication bias

Funnel plots of target lesion failure in bioresorbable and drug-eluting was performed. All studies are included in the plot. The results showed that the funnel plot had medium symmetry and little publication bias (Fig. 5).

## Discussion

CHD is mainly caused by abnormal lipid metabolism, which leads to accumulation of lipid in intima of arteries, and then causes a series of ischemic symptoms [14, 18]. With the transformation of dietary structure and the acceleration of aging, the incidence of CHD showed a significant rising trend and has become a heavy disease burden to the society. For patients with obstructive CHD, stents implantation is an effective therapy to maintain normal coronary circulation.

DES surface coating of high molecular polymer contains anti-smooth muscle proliferation drugs [19–21]. Contemporary DES has better clinical outcomes than bare-metal stents, but there are still risks of stent stenosis and thrombosis due to persistent inflammation, loss of normal vessel curvature and so on [22, 23]. In view of this, BVS was invented to provided mechanical support like DES for 1 year, followed by complete bio-resorption over several years. Several large RCTs showed BVS was noninferior to DES with respect to symptoms control. However, its safety remains to be established.

In our meta-analysis, BVS had a significantly higher risk of target lesion failure, stent thrombosis and cardiac death than DES at 1 year, which indicated that BVS was not as safe as DES. All these results demonstrated that DES was a better therapy than BVS for coronary revascularization. In fact, the AIDA study and ABSORB III study both demonstrated an increased risk of scaffold thrombosis [24, 25]. BVS is, by design and performance, more thrombogenic than current DES. The reasons for higher rate of thrombosis with BVS were not fully clear and some concerns have been raised about the optimal preparation of the lesion and insufficient post-dilatation [26]. In addition, the latest guidelines on coronary revascularization does not support the use of BVS with a class III level of evidence C recommendation [27]. Therefore, interventionalist should be aware of the possible risks related to the use of BVS.

Some limitations existed in this research. First, the present number of studies on BVS is still limited especially in outcome analysis. Second, most of the studies included were investigating ‘Absorb BVS’ device. New BVS with a smaller footprint, less thrombogenicity (e.g., magnesium), faster reabsorption and advanced mechanical properties is under development. We cannot dismiss the safety and efficacy of new BVS technology.

## Conclusion

BVS had a significantly higher risk of target lesion failure, stent thrombosis and cardiac death than DES. DES is a safer treatment strategy than BVS for coronary revascularization.

## Data Availability

Not applicable.

## Abbreviations

BVS: Bioresorbable vascular stents
CHD: Coronary heart disease
DES: Drug-eluting stents
